# Integrated Genetic Networks and Epigenetic Regulation inTooth Development and Maturation

**DOI:** 10.3390/cells15070618

**Published:** 2026-03-30

**Authors:** Dong-Joon Lee, Hyung-Jin Won, Jeong-Oh Shin

**Affiliations:** 1 Department of Oral Histology, College of Dentistry, Dankook University, Cheonan 31116, Republic of Korea; dongjoon@dankook.ac.kr; 2 Institute of Tissue Regeneration Engineering (ITREN), Dankook University, Cheonan 31116, Republic of Korea; 3 Department of Anatomy, College of Medicine, Kangwon National University, Chuncheon 24341, Republic of Korea; hjwon@kangwon.ac.kr

**Keywords:** tooth development, odontogenesis, signaling pathways, epigenetic regulation, DNA methylation, histone modifications, microRNA, dental stem cells, regenerative dentistry

## Abstract

**Highlights:**

**What are the main findings?**

- Tooth development is orchestrated by an integrated network where four conserved signaling pathways (Wnt/β-catenin, BMP, FGF, Shh) dynamically interact with transcription factors across all morphological stages.
- Epigenetic mechanisms, including DNA methylation, histone modifications (e.g., EZH2-mediated H3K27me3), and microRNAs, provide essential fine-tuning for dental stem cell fate determination and differentiation.

**What are the implications of the main findings?**

- Integrating genetic signaling and epigenetic regulatory layers into a unified framework provides a more comprehensive understanding of both normal odontogenesis and the etiology of congenital dental anomalies.
- This systems-level knowledge establishes a crucial foundation for advancing regenerative dentistry, bridging the gap between basic developmental biology and emerging tissue engineering applications.

**Abstract:**

Tooth development or odontogenesis is a complex morphogenetic process that requires tightly regulated interactions between the oral epithelium and mesenchyme of neural crest origin. In this narrative review, we compile existing knowledge regarding gene regulatory networks and epigenetic factors throughout tooth development from initiation to eruption. Signaling between the epithelium and mesenchyme is mediated by four conserved pathways—Wnt/β-catenin, bone morphogenetic protein (BMP), fibroblast growth factor (FGF), and Sonic hedgehog (Shh)—which operate iteratively and interact through extensive crosstalk at each developmental stage. Transcription factors, such as PAX9, MSX1, PITX2, and LEF1, interpret these signals to control cell fate decisions and differentiation. Epigenetic modifications, including DNA methylation, histone modifications, and microRNA-mediated regulation, provide additional layers of control that fine-tune gene expression programs. Unlike existing reviews that address these regulatory mechanisms separately, here we integrate signaling pathways, transcription factor networks, epigenetic regulation, human genetic disorders, dental stem cell biology, and recent single-cell transcriptomic insights into a unified framework. We discuss opportunities to apply developmental biology knowledge towards regenerative dentistry goals, including iPSC-derived dental models and spatially resolved multi-omics approaches, while acknowledging the considerable gap between preclinical findings and clinical applications.

## 1. Introduction

Tooth development represents one of the most well-characterized models of organogenesis, exemplifying the fundamental principles of epithelial–mesenchymal interactions that govern ectodermal appendage formation [1,2]. This highly orchestrated process, termed odontogenesis, involves sequential and reciprocal signaling between the oral epithelium and neural crest-derived ectomesenchyme, resulting in the precise formation of teeth with species-specific morphologies and positions [3,4].

Understanding tooth development extends beyond basic developmental biology. Teeth have essential functions in mastication, speech, and facial esthetics. Congenital dental anomalies, including tooth agenesis (hypodontia, oligodontia, and anodontia) and supernumerary teeth, affect 2–10% of the population, depending on the specific conditions [5,6]. These anomalies result from mutations in genes encoding signaling molecules or transcription factors, providing important insights into the genetic architecture of tooth development.

Remarkable progress has been made in elucidating the molecular mechanisms underlying tooth development. Studies utilizing mouse genetic models have identified numerous essential genes and revealed complex signaling networks [7,8]. Four major signaling pathways, Wnt/β-catenin, BMP, FGF, and Shh, have emerged as central regulators that operate in an iterative and interconnected fashion throughout multiple developmental stages [9,10]. These pathways function through extensive crosstalk and dosage-dependent mechanisms, enabling precise spatial and temporal control of gene expression.

Research has expanded to encompass the epigenetic landscape of tooth development. Epigenetic mechanisms, including DNA methylation, histone modifications, and noncoding RNA regulation, provide additional control over gene expression, which is essential for proper cellular differentiation [11,12]. These modifications enable cells to respond dynamically to developmental cues while maintaining stable differentiation states.

Notably, although individual signaling pathways and epigenetic mechanisms have been extensively studied in isolation, a comprehensive understanding of how these regulatory layers interact to orchestrate the full complexity of odontogenesis remains incomplete. Recent advances in single-cell transcriptomics and spatial genomics have begun to reveal previously unappreciated heterogeneity within dental cell populations, challenging simplified models of linear signaling cascades [9,10]. This review aims to bridge these knowledge gaps by integrating genetic and epigenetic perspectives into a unified framework.

The clinical relevance of tooth developmental biology has increased with advances in regenerative medicine. Understanding molecular and cellular mechanisms provides a foundation for regenerating functional teeth [13,14]. Dental stem cells, including dental pulp stem cells (DPSCs) and periodontal ligament stem cells (PDLSCs), have emerged as promising cellular sources for regenerative applications [15,16]. However, most evidence supporting regenerative approaches has derived from in vitro and animal model studies, with limited clinical trial data available to date.

This review provides a comprehensive examination of the genetic and epigenetic regulation of tooth development from initiation through eruption. We synthesize current knowledge from basic science research, primarily conducted in murine models, and discuss the potential clinical implications while acknowledging the significant translational gaps that remain. By integrating genetic signaling networks with epigenetic regulatory mechanisms, we aim to provide a framework for understanding tooth development that may inform future regenerative strategies. Notably, while numerous reviews have been published between 2023 and 2025 that have addressed individual aspects of tooth development—signaling pathway reviews (e.g., BMP in root development, DPSC signaling cascades) largely ignore epigenetic regulation, whereas epigenetic reviews focus narrowly on dental stem cell differentiation without integrating developmental signaling pathways. Similarly, genetic disorder reviews and single-cell transcriptomic analyses remain disconnected from both categories. No existing review simultaneously integrates all six topics covered here: (1) signaling pathways, (2) transcription factors, (3) epigenetic regulation across multiple mechanisms, (4) genetic disorders, (5) dental stem cells, and (6) single-cell transcriptomics. This review addresses this gap by providing a unified framework that connects these regulatory layers.

This review adopted a narrative approach, with the literature identified through systematic searches of PubMed and Google Scholar using terms including odontogenesis, tooth development, dental epigenetics, dental stem cells, Wnt BMP FGF Shh signaling, and related keywords. Studies published primarily between 2015 and 2025 were prioritized, with seminal earlier works included for their foundational importance. Inclusion was based on relevance to the six integrated topics covered: signaling pathways, transcription factors, epigenetic regulation, genetic disorders, dental stem cells, and single-cell transcriptomics.

## 2. Morphological Stages of Tooth Development

**Figure 1:**
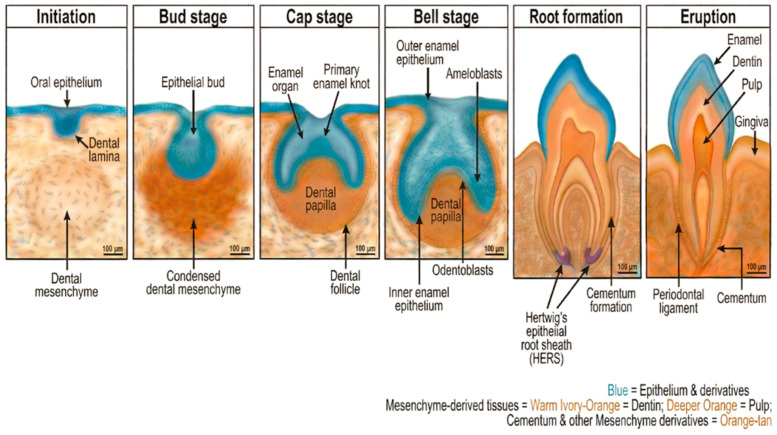
Morphological stages of tooth development. Schematic overview of the six consecutive stages of odontogenesis from initiation to eruption. Initiation: The oral epithelium (blue) thickens to form the dental lamina, which invaginates into the underlying neural crest-derived dental mesenchyme (orange/tan). Bud stage: The epithelial bud invades condensed dental mesenchyme. Cap stage: the enamel organ develops with the primary enamel knot as a transient signaling center; the dental papilla and dental follicle (dental sac) become morphologically distinct. Bell stage: Histodifferentiation gives rise to ameloblasts (from the inner enamel epithelium) and odontoblasts (from dental papilla cells); the outer enamel epithelium is distinctly identifiable. Root formation: Hertwig’s epithelial root sheath (HERS) directs apical root elongation and induces odontoblast differentiation, resulting in the deposition of pulp/dentin; cementum formation begins along the root surface. Eruption: The mature tooth erupts through the gingiva and alveolar bone, displaying enamel, dentin, pulp, gingiva, cementum, periodontal ligament, and alveolar bone. Color code: blue = epithelium and derivatives; warm ivory-orange = dentin; deeper orange = pulp; cementum and other mesenchyme derivatives retain existing colors. Scale bars = 100 μm.

Tooth development proceeds through well-defined morphological stages, each characterized by distinct structural features and molecular signatures (Figure 1). These stages—initiation, bud, cap, bell, and maturation—represent the progressive transformation of epithelial thickenings into complex mineralized organs [7,17].

### 2.1. Initiation Stage

Tooth development initiation occurs during the sixth week of human embryonic development (approximately embryonic day 11 in mice), when the oral ectoderm thickens at specific locations along presumptive dental arches [2]. This thickening, the dental lamina, forms a continuous band of stratified epithelium, giving rise to individual tooth germs. The positioning along the oral-aboral axis is determined by the combinatorial expression of homeobox genes, including Msx, Dlx, Barx, and Pitx families, establishing the “odontogenic code” [18].

Within the dental lamina, localized epithelial thickenings called placodes mark individual tooth formation sites. Placode formation involves complex signaling interactions, with Wnt, BMP, and FGF signals playing essential roles in specifying positions and initiating the morphogenetic cascade [19,20]. PITX2 is among the earliest markers of the dental placode epithelium and is required for subsequent development [21].

### 2.2. Bud Stage

The bud stage is characterized by the invasion of the dental placode into the underlying neural crest-derived ectomesenchyme, forming a distinct epithelial bud surrounded by condensed mesenchymal cells [3]. This morphogenetic movement involves coordinated cell proliferation, migration, and cytoskeletal rearrangements.

A critical event is the transfer of odontogenic potential from the epithelium to the mesenchyme. Although the dental epithelium initially possesses tooth-inducing capacity, this potential shifts to the mesenchyme by the late bud stage [1]. This transfer is mediated by signaling molecules secreted by the epithelium, including BMPs, FGFs, and Wnts, which activate key transcription factors, such as PAX9, MSX1, and LEF1, in the mesenchyme [22,23].

### 2.3. Cap Stage

The bud-to-cap transition represents a pivotal moment, marked by the formation of the primary enamel knot (pEK)—a transient signaling center at the tip of the epithelial invagination [3]. The cap-stage enamel organ consists of the outer enamel epithelium, inner enamel epithelium surrounding the enamel knot, and the stellate reticulum. The underlying condensed mesenchyme differentiates into the dental papilla (forming the pulp and dentin) and dental follicle (forming periodontal tissues).

The enamel knot functions as an organizing center, patterning the developing tooth through the secretion of Shh, BMPs, FGFs, and Wnts [24]. These signals regulate cell proliferation in the surrounding cervical loop epithelium, whereas enamel knot cells remain quiescent and eventually undergo apoptosis.

### 2.4. Bell Stage

The bell stage is named for the bell-shaped enamel organ, which expands and encloses the dental papilla. This stage features extensive histodifferentiation, in which the inner enamel epithelium and dental papilla precursor cells differentiate into ameloblasts and odontoblasts, respectively [7].

In multicuspid teeth, secondary enamel knots form at future cusp positions, establishing complex occlusal morphology. The timing and location of secondary enamel knot formation, regulated by an activator–inhibitor signal interplay, determine cusp number and arrangement [3]. This cusp patterning process is explained by the activator–inhibitor (Turing-type) model, in which activating signals (e.g., BMPs and FGFs) from secondary enamel knots promote cusp growth, whereas lateral inhibition by antagonists (e.g., SOSTDC1 and follistatin) prevents adjacent cusp formation, thereby establishing species-specific spacing and number of cusps [3,24].

Odontoblasts secrete a predentin matrix containing type I collagen and non-collagenous proteins, such as dentin sialophosphoprotein (DSPP) and dentin matrix protein 1 (DMP1). Ameloblasts secrete enamel matrix proteins, including amelogenin, ameloblastin, and enamelin, which serve as templates for hydroxyapatite crystal formation.

### 2.5. Root Formation and Maturation

After crown formation, root development proceeds through Hertwig’s epithelial root sheath (HERS), a bilayered epithelial structure derived from the cervical loop [25,26]. HERS proliferates apically, guiding root elongation and inducing root odontoblast differentiation. Subsequently, HERS fenestrates, allowing dental follicle cells to contact the root surface and differentiate into cementoblasts.

The molecular mechanisms governing root formation involve Wnt/β-catenin, BMP, and TGF-β signaling with distinct spatiotemporal patterns [10]. Transcription factors, including nuclear factor I-C (NFIC), have been identified as regulators specific to root development. Recent studies have demonstrated that BMP/SMAD signaling exhibits dynamic activity during root morphogenesis, progressively shifting from crown to root regions during postnatal development [27].

## 3. Genetic Regulation: Signaling Pathways

**Figure 2:**
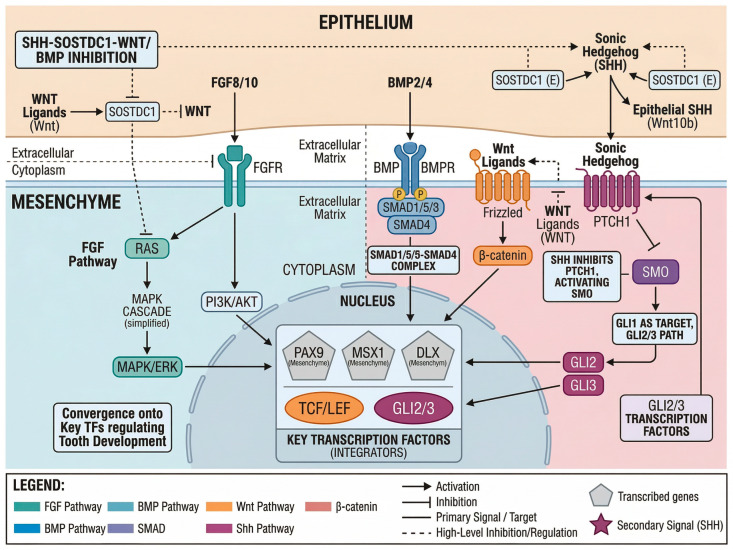
Integrated signaling pathway network in tooth development. Schematic overview illustrating the four major signaling pathways and their crosstalk during epithelial–mesenchymal interactions in odontogenesis: Wnt/β-catenin, bone morphogenetic protein (BMP), fibroblast growth factor (FGF), and Sonic hedgehog (Shh). The diagram is divided into epithelial (left, orange background) and mesenchymal (right, blue background) compartments, with the nucleus (gray) depicted centrally. Wnt pathway (orange circle icons): Wnt ligands bind to Frizzled receptors and low-density lipoprotein receptor-related protein 5/6 (LRP5/6) co-receptors, inhibiting glycogen synthase kinase 3β (GSK-3β)-mediated β-catenin phosphorylation, leading to nuclear translocation and transcriptional activation via T-cell factor/lymphoid enhancer factor (TCF/LEF) family transcription factors (e.g., LEF1). BMP pathway (blue triangle icons): BMP2/4 ligands bind to BMP receptors (BMPRs), leading to SMAD1/5/8 phosphorylation and complex formation with SMAD4, allowing nuclear translocation and target gene regulation. FGF pathway (blue square icons): FGF8/10 ligands bind to FGF receptors (FGFRs) and activate the phosphoinositide 3-kinase (PI3K)/AKT and mitogen-activated protein kinase (MAPK)/extracellular signal-regulated kinase (ERK) cascades. Shh pathway (red star icons): Shh binds to Patched 1 (PTCH1), relieving inhibition of Smoothened (Smo) and allowing GLI2/3 transcription factor activation; GLI1 functions as a transcriptional target of the pathway while GLI2/3 serve as the primary signal transducers. In the mesenchyme, key transcription factors (PAX9, MSX1, and DLX; hexagonal icons) integrate these signaling inputs. The Shh–Sostdc1–Wnt/BMP inhibitory feedback connection is mediated by sclerostin domain-containing protein 1 (Sostdc1), which is expressed in the epithelium. Arrows indicate activation; flat-ended lines indicate inhibition; solid lines indicate translocation to the nucleus; and dashed lines indicate secondary branches or feedback loops.

Morphogenetic events in tooth development are orchestrated by conserved signaling pathways that operate iteratively throughout multiple stages (Figure 2). The Wnt, BMP, FGF, and Shh pathways form an interconnected regulatory network characterized by extensive crosstalk and dosage sensitivity [9,10]. Evidence for pathway functions primarily derives from mouse genetic models, and direct evidence in human tooth development remains limited.

Importantly, these four pathways do not function in isolation but rather form a highly integrated signaling network. Hermans et al. (2021) demonstrated that canonical Wnt and Shh signaling engage in reciprocal regulation, with Wnt activation promoting Shh expression in the enamel knot, while Shh-induced Sostdc1 feeds back to inhibit Wnt signaling [9]. This give-and-take mechanism ensures balanced proliferation and differentiation, and its disruption leads to predictable dental phenotypes in mouse models. Understanding these pathway interactions, rather than individual pathway functions alone, is essential for developing targeted therapeutic strategies.

### 3.1. Wnt/β-Catenin Signaling

The Wnt/β-catenin pathway plays fundamental roles throughout tooth development, from placode specification to root formation [10,20]. Wnt ligands, particularly WNT10A and WNT10B, are expressed in the dental epithelium during early stages, activating canonical β-catenin signaling essential for placode formation. Upon Wnt ligand binding to Frizzled receptors and LRP5/6 co-receptors, the destruction complex containing APC, Axin, and GSK3β is inhibited, allowing β-catenin nuclear translocation. In the nucleus, β-catenin interacts with Lef1/TCF transcription factors to activate target genes, including Shh and Lef1 itself [28]. This regulatory mechanism operates through TCF/LEF1 binding sites in long-range enhancers that are active during oral development. Epithelial overexpression of the Wnt inhibitor DKK1 arrests tooth development at the bud stage in transgenic mice [19]. Conversely, constitutive β-catenin activation leads to ectopic Shh expression and supernumerary teeth [29]. LEF1 knockout mice exhibit arrested development at the bud stage because LEF1 directly activates FGF4 in the enamel knot [28]. In humans, WNT10A mutations are a major cause of hypodontia and ectodermal dysplasia, supporting the translational relevance of these findings [5]. Andl et al. [19] demonstrated Wnt requirement in ectodermal appendage development including teeth; subsequent tooth-specific studies by Chen et al. [20] confirmed this essential role in odontogenic mesenchyme.

Furthermore, polymorphisms in AXIN2, a key negative regulator of the Wnt/β-catenin pathway, have been identified as risk factors for selective tooth agenesis, reinforcing the critical role of precise Wnt signaling in human dental development [30].

### 3.2. Bone Morphogenetic Protein (BMP) Signaling

BMP signaling contributes at multiple stages by regulating epithelial–mesenchymal interactions and cell differentiation [17]. BMP2 and BMP4 are expressed in complex spatiotemporal patterns and signal through BMPR1A to activate Smad1/5/8-mediated transcription.

During initiation, BMP4 expression in the presumptive dental mesenchyme helps pattern the odontogenic field. BMP4 can induce MSX1 and MSX2 expression in the mesenchyme, which in turn regulates BMP4 via a positive feedback loop. This interaction is modulated by the BMP inhibitor SOSTDC1 (also known as WISE), which provides spatial restriction and inhibits Wnt signaling by binding to LRP co-receptors [31]. Recent studies have demonstrated that BMP/SMAD signaling exhibits dynamic spatiotemporal activity during root morphogenesis. Between postnatal days 3–5 in mice, concurrent with root initiation, stem cells crucial for root development emerge alongside evident BMP/SMAD signaling within the dental pulp [27]. Disruption of BMPR1A in root mesenchyme results in the complete absence of molar roots despite successful crown development, indicating that BMP signaling requirements differ between crown and root formation.

### 3.3. Fibroblast Growth Factor (FGF) Signaling

FGF signaling, primarily through FGFR1 and FGFR2, is essential for morphogenesis and proliferation [17]. Multiple FGF ligands, including FGF3, FGF4, FGF8, FGF9, FGF10, and FGF20, are expressed in distinct patterns during tooth development. FGF8, expressed early in the presumptive dental epithelium, is necessary and sufficient to induce mesenchymal PAX9 expression. At the bud stage, FGF signaling maintains mesenchymal competence for continued development. In the enamel knot, FGF4 secretion stimulates cervical loop proliferation, driving morphogenetic expansion [28]. Mutations in FGF signaling components have been associated with various dental anomalies in humans, although the precise genotype-phenotype correlations remain to be fully established [5].

### 3.4. Sonic Hedgehog (Shh) Signaling

The Shh pathway coordinates epithelial morphogenesis, with Shh expression marking the enamel knot from the late-bud stage [24,32]. Shh signals through Patched (PTCH1), relieving Smoothened (Smo) inhibition and enabling Gli transcription factor activation. Shh signaling promotes epithelial proliferation and is required for proper bud-to-cap invagination. Conditional Shh or Smo deletion results in arrested development with hypoplastic teeth [32]. A critical regulatory interaction involves Shh-induced SOSTDC1 expression, creating a negative feedback loop that inhibits epithelial Wnt signaling [24,31]. This bidirectional regulation between the Wnt and Shh pathways is crucial for maintaining developmental precision.

### 3.5. Pathway Crosstalk and Feedback Regulation

A defining feature of odontogenesis is that Wnt, BMP, FGF, and Shh do not operate as isolated linear cascades; rather, they form an interdependent control network shaped by reciprocal regulation, transient signaling centers (e.g., the enamel knot), and local inhibitory feedback. For example, epithelial Shh is required for bud-to-cap morphogenesis and is tightly coupled to Wnt output: LEF1 directly links canonical Wnt activity to Shh regulatory elements, while Shh can induce the secreted modulator SOSTDC1, which in turn dampens epithelial Wnt signaling, creating a self-limiting feedback loop [24,28,32]. Similarly, pathway-specific inhibitors (e.g., DKK1 for Wnt) can halt progression when ectopically expressed, emphasizing the dose- and stage-dependence of signaling thresholds during placode/bud formation [19]. These feedback architectures likely underlie robust patterning (cusp number/position, cervical loop maintenance, and tissue boundary formation); however, they remain incompletely mapped at the cell-type resolution in vivo—an important gap that multi-omic and spatial approaches are now poised to address [33,34].

### 3.6. Additional Regulatory Modules and Mechanical Cues

Although Wnt/β-catenin, BMP, FGF, and Shh constitute the best-characterized morphogenetic circuits in odontogenesis, multiple additional pathways and physical cues modulate tooth patterning, epithelial–mesenchymal reciprocity, and lineage commitment. These modules often interface directly with the core four-pathway network and can help explain phenotypes (and regenerative responses) that are not readily captured by the Wnt/BMP/FGF/Shh framework.

EDA/EDAR/NF-κB axis: Ectodysplasin (EDA)–EDAR signaling (TNF superfamily) regulates the size and activity of epithelial signaling centers (enamel knots), thereby influencing cusp patterning and, in some contexts, tooth number. In murine tooth organogenesis, EDA/EDAR signaling is positioned at the intersection of canonical Wnt output and mesenchymal TGF-β family cues, and pathway dosage effects can produce opposite crown phenotypes (reduced vs. increased cusp complexity). Downstream targets include Fgf-family components, such as Fgf20, consistent with an activator–inhibitor balance model of cusp morphogenesis [35,36,37,38].

Notch pathway: Notch signaling contributes to epithelial cell fate decisions during ameloblast lineage progression and has also been mechanistically linked to crown morphogenesis. Importantly, BMP and FGF inputs can modulate Jag2–Notch activity in the developing tooth germ, thereby providing a route by which morphogen gradients are transduced into discrete epithelial differentiation programs [39,40].

Hippo–YAP/TAZ and mechanobiology: The Hippo pathway effectors (YAP/TAZ) provide a mechanosensitive link between epithelial architecture and the establishment of developmental signaling centers. Functional studies implicate YAP in primary enamel knot regulation and cusp patterning, and αE-catenin-mediated suppression of YAP/TAZ is required for proper signaling center formation during tooth development. These data support a model in which tissue mechanics and junctional context tune morphogenetic programs along with canonical ligand–receptor pathways [41,42].

Retinoic acid (RA) signaling: Retinoic acid (RA) signaling has long been recognized as a dose- and stage-dependent modulator of craniofacial and dental patterning. Classic organ culture studies have demonstrated that exogenous RA can perturb murine incisor morphogenesis, consistent with RA-mediated shifts in epithelial–mesenchymal patterning and differentiation programs. In contemporary regenerative contexts, RA should be treated as a potentially powerful but context-sensitive lever, warranting careful titration and cell-type-resolved readouts [43].

## 4. Genetic Regulation: Transcription Factors

Activation of the signaling pathway converges on transcription factors that execute stage-specific gene expression programs (Table 1). These factors integrate multiple signaling inputs and regulate downstream target genes essential for cell fate determination [6,44].

### 4.1. PAX9

PAX9 is a paired-box transcription factor expressed in dental mesenchyme from early initiation and plays essential roles in tooth development [22,45]. Pax9-deficient mice lack all teeth, with developmental arrest at the bud stage, demonstrating its critical requirement for progression beyond early stages.

In humans, PAX9 mutations represent the most common cause of nonsyndromic tooth agenesis [45]. A systematic review by Intarak et al. (2023) comprehensively analyzed PAX9 variant profiles and associated tooth agenesis patterns, confirming that the second molar is most consistently affected, whereas the mandibular first premolar is least affected [45]. Mutation severity correlates with phenotype severity: nonsense mutations produce more severe phenotypes affecting multiple teeth, whereas missense mutations result in milder effects with fewer missing teeth.

### 4.2. MSX1 and MSX2

MSX1 acts synergistically with PAX9 in the dental mesenchyme [23,46]. Msx1-deficient mice exhibit arrested tooth development and cleft palate. MSX1 functions downstream of BMP signaling and participates in feedback loops that maintain odontogenic competence. The PAX9-MSX1 synergistic relationship is mechanistically important, as their proteins interact to synergistically activate downstream genes involved in dental organogenesis. Combined mutations can result in more severe agenesis than either mutation alone, suggesting a cooperative function in establishing the odontogenic program.

### 4.3. PITX2

PITX2 is a bicoid-related homeobox transcription factor expressed in the dental epithelium from the earliest stages [21]. It represents the earliest marker of odontogenic potential and regulates cell proliferation and epithelial–mesenchymal signaling. PITX2 mutations cause Axenfeld–Rieger syndrome, characterized by eye, craniofacial, and dental abnormalities, including tooth agenesis and microdontia. This clinical syndrome provides direct evidence of the role of PITX2 in human dental development.

### 4.4. LEF1

LEF1 (lymphoid enhancer-binding factor 1) is the primary mediator of canonical Wnt signaling during tooth development [28]. Lef1 knockout mice show arrested development at the bud stage. Mechanistically, LEF1 directly activates FGF4 transcription in the enamel knot and binds to SHH enhancers, thereby enabling direct Wnt regulation of Shh expression. This positions LEF1 as a critical node integrating Wnt signaling with downstream FGF and Shh pathways.

## 5. Epigenetic Regulation of Tooth Development

**Figure 3:**
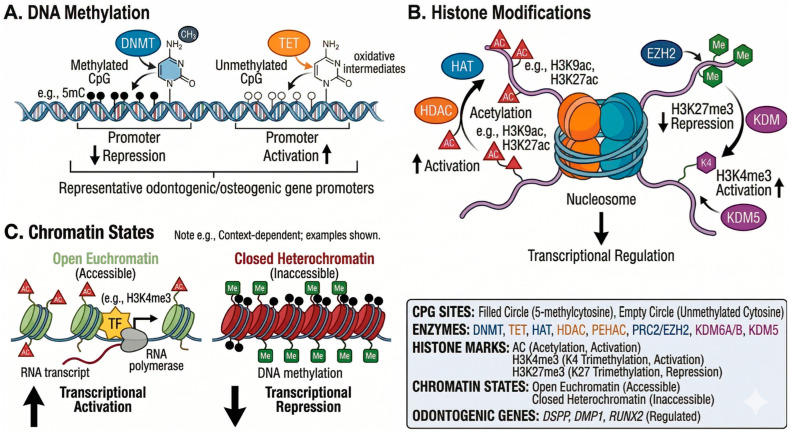
Epigenetic regulatory mechanisms in odontogenic differentiation. (**A**) DNA methylation: DNA methyltransferases (DNMTs; blue) add a methyl group (CH3) to cytosine residues at CpG dinucleotides, producing 5-methylcytosine (5mC) and promoting transcriptional repression. Ten-eleven translocation (TET) family enzymes (orange) catalyze oxidative demethylation of 5mC through multiple oxidative intermediates, resulting in promoter activation. Representative regulated odontogenic/osteogenic gene promoters include dentin sialophosphoprotein (DSPP), dentin matrix protein 1 (DMP1), and runt-related transcription factor 2 (RUNX2). (**B**) Histone modifications: Histone acetyltransferases (HATs; green) add acetyl groups (AC) to lysine residues on histone tails (e.g., H3K9ac, H3K27ac), promoting open chromatin configuration and transcriptional activation. Histone deacetylases (HDACs; orange) remove acetyl groups, leading to chromatin compaction and transcriptional silencing. Enhancer of zeste homolog 2 (EZH2; a component of the polycomb repressive complex 2, PRC2) catalyzes trimethylation of histone H3 at lysine 27 (H3K27me3), resulting in transcriptional repression. Lysine demethylases (KDM) remove methyl marks: KDM6A/B removes H3K27me3 repressive marks to enable activation, while KDM5 removes H3K4me3 activation marks. (**C**) Chromatin states: Open euchromatin (left) is characterized by acetylated histones (e.g., H3K4me3), permitting transcription factor (TF) binding and RNA polymerase recruitment for transcriptional activation (e.g., RUNX2, DSPP, DMP1). Closed heterochromatin (right) is associated with methylated DNA (Me) and tightly packed nucleosomes, rendering chromatin inaccessible and resulting in transcriptional repression (e.g., RUNX2, DSPP, DMP1). Legend box: CpG sites are represented by filled circles (5-methylcytosine) and empty circles (unmethylated cytosine). Enzymes are color-coded as follows: DNMT (blue), TET (orange), HAT (green), HDAC (orange), p300/CBP-associated HAC (PEHAC), PRC2/EZH2, KDM6A/B, and KDM5. Histone marks: AC (acetylation, activation), H3K4me3 (K4 trimethylation, activation), and H3K27me3 (K27 trimethylation, repression). CHROMATIN STATES: ‘Open euchromatin’ denotes transcriptionally permissive chromatin with acetylated histones and accessible DNA; ‘closed heterochromatin’ denotes transcriptionally repressive chromatin with methylated DNA and compacted nucleosomes. ODONTOGENIC GENES shown include DSPP (dentin sialophosphoprotein), DMP1 (dentin matrix protein 1), and RUNX2 (runt-related transcription factor 2), which serve as representative markers of odontoblastic differentiation regulated by the epigenetic mechanisms depicted.

In addition to GRNs, epigenetic mechanisms provide additional control that is essential for proper tooth development (Figure 3). DNA methylation, histone modification, and noncoding RNA regulation enable precise spatiotemporal control of gene expression [11,12]. It is important to note that most evidence for epigenetic regulation in dental contexts has derived from in vitro studies using dental stem cells, with limited in vivo validation.

The interplay between genetic signaling pathways and epigenetic modifications represents a critical but incompletely understood aspect of odontogenesis. For instance, Wnt/β-catenin signaling can modulate the activity of the histone methyltransferase EZH2, which in turn affects chromatin accessibility at developmental gene loci [11]. Similarly, BMP signaling influences DNA methylation patterns by regulating DNMT expression. These bidirectional interactions suggest that genetic and epigenetic regulatory layers are not simply additive but form a deeply integrated network.

### 5.1. DNA Methylation

DNA methylation, which occurs at CpG dinucleotides through DNA methyltransferases (DNMTs), plays a central role in regulating odontogenic differentiation [12,47]. Promoter methylation typically silences gene expression by preventing transcription factor binding. During DPSC odontoblast differentiation in culture, dynamic methylation changes regulate key transcription factors. KLF4 undergoes promoter demethylation during differentiation, enabling its upregulation and subsequent activation of odontogenic genes [12]. The TET family of DNA demethylases plays important roles through active demethylation. TET1 promotes odontogenic differentiation by regulating FAM20C demethylation; TET1 knockdown in cultured DPSCs inhibits both proliferation and differentiation [12]. DNA methyltransferase inhibitors can promote differentiation by upregulating DSPP and DMP1 expression in in vitro systems. In PDLSCs, lower methylation of osteogenic genes, including RUNX2, osteopontin, and ALP, correlates with enhanced bone formation capacity in culture and xenograft models [47]. These findings suggest potential therapeutic applications but require validation in clinical settings. Specifically, 5-Aza-2′-deoxycytidine (5-Aza-dC, decitabine), a well-characterized DNMT inhibitor, has been shown to demethylate odontogenic gene promoters and enhance mineralization in DPSC cultures.

### 5.2. Histone Modifications

Histone modifications regulate chromatin accessibility through the dynamic addition or removal of chemical groups [11]. Acetylation, mediated by histone acetyltransferases (HATs), including p300, promotes gene expression by loosening chromatin. In contrast, histone deacetylases (HDACs) remove acetyl groups, promoting chromatin compaction and gene silencing.

Histone methylation shows context-dependent effects; H3K4me3 typically marks active promoters, whereas H3K27me3 is associated with transcriptional repression. Several histone demethylases regulate odontogenic differentiation [11,12]:

- KDM6B: Decreases H3K27me3 levels at the IGFBP5 promoter, promoting odontoblast differentiation and mineralization in PDLSCs (in vitro studies).
- KDM2A: Suppresses differentiation by reducing H3K4 and H3K36 methylation at the epiregulin (EREG) promoter.
- KDM4B: Upregulated by DLX5, KDM4B promotes osteo-/dentinogenesis in stem cells from apical papillae (SCAPs) in nude mouse xenograft models.

Histone methyltransferase EZH2, which catalyzes H3K27 trimethylation, plays a critical role in modulating Wnt/β-catenin signaling [11]. EZH2 reduction leads to decreased H3K27me3 levels, resulting in β-catenin accumulation and Wnt pathway activation, thereby promoting osteogenesis and dentinogenesis in in vitro differentiation assays. Mechanistically, EZH2 directly increases H3K27me3 levels on the promoters of Wnt1, Wnt6, and Wnt10a, thereby silencing Wnt gene transcription and suppressing canonical Wnt/β-catenin signaling [48]. Among these Wnt ligands, Wnt10a has been shown to play a particularly important role in dentinogenesis: Wnt10a stimulates the differentiation of dental pulp cells into odontoblasts and promotes the formation of mineralized dentin nodules in three-dimensional culture systems [49]. Furthermore, in vivo studies have demonstrated that conditional deletion of Ezh2 in the dental mesenchyme disrupts molar root patterning and furcation formation, underscoring the essential role of EZH2-mediated epigenetic regulation in tooth morphogenesis beyond osteogenesis [50]. Collectively, these findings indicate that EZH2 serves as a key epigenetic gatekeeper that regulates both osteogenic and dentinogenic differentiation through H3K27me3-mediated repression of multiple Wnt ligands.

### 5.3. MicroRNA Regulation

**Figure 4:**
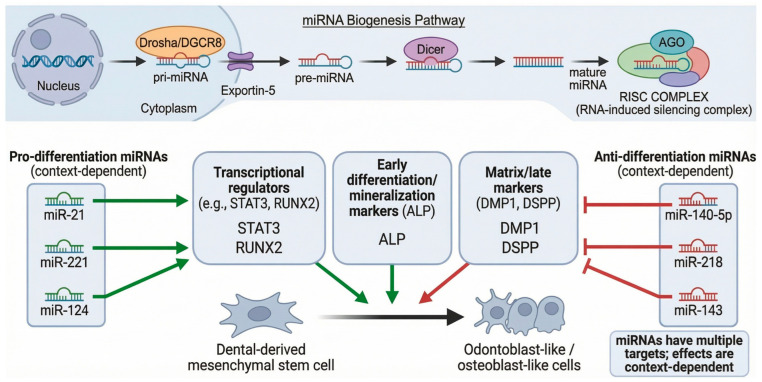
Role of miRNAs in odontogenic differentiation. (Upper panel): Simplified diagram depicting microRNA (miRNA) biogenesis. Primary miRNA (pri-miRNA) is transcribed from DNA in the nucleus and processed by the Drosha/DiGeorge syndrome critical region 8 (DGCR8) microprocessor complex into precursor miRNA (pre-miRNA). Exportin-5 transports pre-miRNA from the nucleus to the cytoplasm, where it is cleaved by Dicer into mature miRNAs. Mature miRNAs are loaded into Argonaute (AGO) protein within the RNA-induced silencing complex (RISC) for target mRNA recognition and post-transcriptional regulation. (Lower panel): Functional classification of miRNAs that regulate dental-derived mesenchymal stem cell differentiation into odontoblast-like/osteoblast-like cells. Pro-differentiation miRNAs (left, green arrows = promotion; context-dependent): miR-21 promotes odontoblast differentiation via the signal transducer and activator of transcription 3 (STAT3) signaling axis; miR-221 enhances odontogenic gene expression; and miR-124 promotes mineralization. These miRNAs positively regulate key transcriptional regulators (e.g., STAT3 and RUNX2), early differentiation/mineralization markers (alkaline phosphatase, ALP), and matrix/late markers (dentin matrix protein 1, DMP1, and dentin sialophosphoprotein, DSPP). Anti-differentiation miRNAs (right, red flat-ended lines = inhibition; context-dependent): miR-140-5p suppresses differentiation markers; miR-218 downregulates DMP1 and DSPP expression; and miR-143 inhibits osteogenic markers. Note: In the figure, miR-140-5p is depicted as inhibiting the matrix/late markers box (DMP1 and DSPP), consistent with the downstream effect of miR-140-5p targeting Wnt1/β-catenin signaling, which indirectly reduces DSPP and DMP-1 expression in dental pulp stem cells. miRNAs have multiple targets, and their effects are context-dependent.

MicroRNAs (miRNAs) regulate gene expression post-transcriptionally by binding target mRNAs (Figure 4). Numerous miRNAs regulate tooth development and dental stem cell differentiation [51,52]. A systematic review and bioinformatic analysis by Iranmanesh et al. (2023) identified over 30 miRNAs that regulate odontogenic and osteogenic differentiation of human dental pulp-derived mesenchymal stem cells, providing a comprehensive landscape of miRNA-mediated regulation in dental stem cell biology (Table 2) [51].

Promotive miRNAs (in vitro evidence)

- miR-21: Promotes differentiation by enhancing STAT3 signaling (e.g., a TNF-α–miR-21–STAT3 axis), likely via indirect modulation of STAT3 activity rather than direct STAT3 mRNA targeting; supported by DPSC in vitro studies and transplantation models.
- miR-221 and miR-124: Enhance odontogenic gene expression and mineralization in DPSC cultures. Suppressive miRNAs (in vitro evidence):
- miR-140-5p: Inhibits odontoblastic differentiation by targeting the Wnt1/β-catenin signaling pathway, thereby reducing DSPP and DMP-1 expression.
- miR-218: Suppresses DMP1 and DSPP expression.
- miR-143: Inhibits multiple osteogenic markers.

Bioinformatics analysis revealed that these miRNAs target MAPK, PI3K-Akt, Wnt, and FoxO pathway components [51]. Furthermore, Giovannetti et al. (2024) identified hsa-let-7a-5p and hsa-miR-103a-3p as targeting tooth anomaly genes, KREMEN1 and PIK3R1, which are enriched in TGF-β/Wnt signaling, providing novel computational insights into miRNA-mediated dental pathology [52]. However, these computational predictions require experimental validation.

It is important to acknowledge that miRNA studies in dental stem cells have shown some conflicting results, with individual miRNAs sometimes exhibiting opposing effects depending on cell type, culture conditions, or experimental methodology. These discrepancies highlight the need for standardized protocols and validation across multiple experimental systems before therapeutic application.

## 6. Root Formation and Tooth Eruption

**Figure 5:**
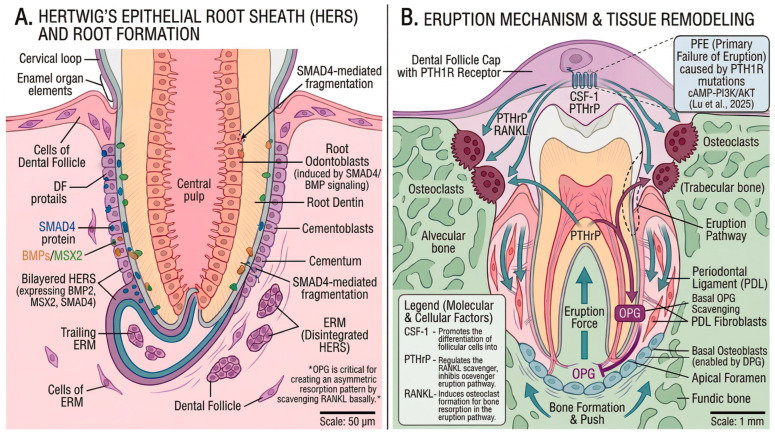
Root formation and tooth eruption. (**A**) Hertwig’s epithelial root sheath (HERS), a transient bilayer derived from the cervical loop, guides root morphogenesis and induces the differentiation of dental papilla cells into root odontoblasts. As root dentin is deposited, HERS undergoes fragmentation, allowing dental follicle-derived mesenchymal cells to contact the root surface and differentiate into cementoblasts, which deposit cementum. Residual HERS cells persist within the periodontal ligament as epithelial rests of Malassez (ERM). BMP2/4, MSX2, and SMAD4-dependent signaling contribute to epithelial-mesenchymal coordination during this process. (**B**) Tooth eruption is coordinated by spatially regulated bone remodeling and periodontal attachment formation. Coronal alveolar bone is resorbed by osteoclasts to create the eruption pathway, whereas osteoblast-mediated bone formation at the fundic region contributes to eruptive movement. The dental follicle regulates this process through CSF-1, asymmetric RANKL/OPG signaling, and PTHrP–PTH1R signaling, while the developing periodontal ligament provides lateral traction. Human PTH1R mutations causing primary failure of eruption (PFE) further support the essential role of this pathway in eruption force generation and are linked to disruption of cAMP–PI3K/AKT signaling.

After crown development, root formation and eruption establish functional dentition (Figure 5). These processes involve distinct molecular mechanisms, although they share some regulatory pathways with earlier stages [33,34].

### 6.1. Hertwig’s Epithelial Root Sheath (HERS)

The HERS is a transient bilayered structure essential for root formation [25,26]. Derived from the cervical loop, HERS performs the following critical functions:

- Root morphogenesis: HERS outlines root shape and number by guiding apical extension.
- Odontoblast induction: HERS induces dental papilla cells to differentiate into root odontoblasts via secreted signaling molecules.
- Cementogenesis: After inducing dentin formation, HERS fragments, allowing dental follicle cells to contact the root surface and differentiate into cementoblasts.

HERS cells express BMP2, BMP4, and MSX2, regulating adjacent tissue differentiation [26]. Following fragmentation, HERS cells persist as epithelial rests of Malassez (ERM) within the periodontal ligament [25]. SMAD4, as a central intracellular mediator of canonical BMP signaling, plays a crucial regulatory role: conditional inactivation of Smad4 in the dental epithelium in mice leads to abnormal dentin formation and shortened roots [27].

### 6.2. Tooth Eruption Mechanisms

Tooth eruption requires coordinated bone resorption to create an eruption pathway, generation of eruption force, and bone formation at the crypt base [33,34]. The dental follicle coordinates eruption through signaling molecules regulating osteoclast and osteoblast activity:

- CSF-1 is essential for osteoclast differentiation and is expressed in the dental follicle overlying the erupting tooth.
- The RANKL/OPG axis: RANKL promotes osteoclastogenesis, and the dental follicle produces RANKL coronally and OPG basally, creating an asymmetric resorption pattern.
- PTHrP regulates the RANKL/OPG balance and is required for eruption through the PTH1R receptor.

Primary failure of eruption (PFE), caused by PTH1R loss-of-function mutations in humans, demonstrates that PTH1R signaling is essential for eruption force generation. Lu et al. (2025) recently identified a novel PTH1R mutation that causes PFE through disruption of the cAMP-PI3K/AKT signaling pathway, further elucidating the molecular mechanisms underlying this condition [59]. This human genetic evidence provides strong support for the mechanistic models developed in animal studies.

## 7. Clinical Implications and Regenerative Applications

**Figure 6:**
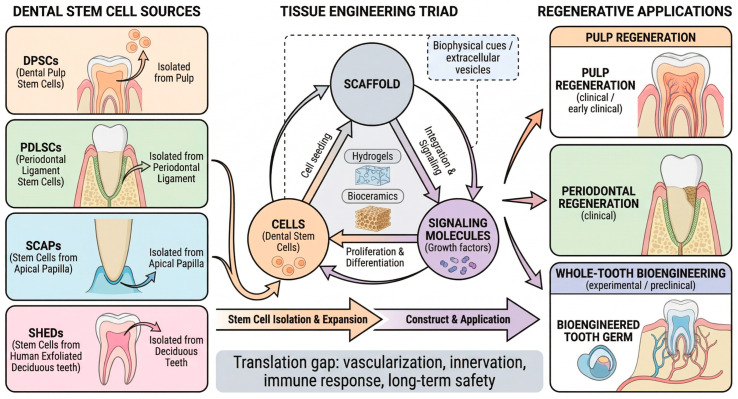
Dental stem cells and clinical applications in regenerative dentistry. (**Left panel**) (Dental stem cell sources): Four main dental stem cell populations are depicted with their respective tissues of origin: dental pulp stem cells (DPSCs), isolated from the pulp of permanent teeth; periodontal ligament stem cells (PDLSCs), isolated from the periodontal ligament; stem cells from the apical papilla (SCAPs), isolated from the apical papilla of developing teeth; and stem cells from human exfoliated deciduous teeth (SHEDs), isolated from the pulp of deciduous teeth. (**Center panel**) (Tissue engineering triad): The three key components of tissue engineering are represented in a circular relationship: cells (dental stem cells) undergo proliferation and differentiation; scaffolds (hydrogels, bioceramics) provide structural support and deliver biophysical cues/extracellular vesicles; and signaling molecules (growth factors) drive cell behavior through integration and signaling. Cell seeding onto scaffolds combined with signaling molecule delivery enables construct assembly and therapeutic applications. (**Right panel**) (Regenerative applications): Three clinical targets at different translational stages: pulp regeneration (clinical/early clinical stage), periodontal regeneration (clinical stage), and whole-tooth bioengineering (experimental/preclinical stage), represented by a bioengineered tooth germ. The bottom bar summarizes the current translation gap, including remaining challenges related to vascularization, innervation, immune response, and long-term safety. Abbreviations: DPSCs, dental pulp stem cells; PDLSCs, periodontal ligament stem cells; SCAPs, stem cells from the apical papilla; SHEDs, stem cells from human exfoliated deciduous teeth.

Understanding genetic and epigenetic mechanisms has potential clinical implications, particularly for the diagnosis of dental anomalies and the advancement of regenerative dentistry (Figure 6) [13,14,60]. However, it is essential to acknowledge that most regenerative strategies are currently in the preclinical stage, with limited clinical trial data available.

### 7.1. Genetic Diagnosis of Dental Anomalies (Established Clinical Applications)

Gene identification enables genetic diagnosis and counseling for affected families [5,45]. This is the most clinically mature application of developmental biology knowledge. Key loci with established clinical utility:

- AXIN2: Wnt pathway regulator; variants/polymorphisms have been associated with selective tooth agenesis in human genetic studies [30].
- PAX9: The most common cause of nonsyndromic oligodontia, affecting primarily the molars.
- MSX1: Causes oligodontia affecting the premolars and third molars.
- WNT10A: A major contributor to hypodontia and ectodermal dysplasia.
- PTH1R: Causes primary failure of eruption.

Genetic testing for these loci is now clinically available and can inform treatment planning and family counseling.

### 7.2. Dental Stem Cells for Regenerative Medicine

Dental tissues harbor multiple stem cell populations with regenerative potential [15,16,61,62].

- DPSCs (Dental Pulp Stem Cells): Capable of odontogenic, neurogenic, and angiogenic differentiation in vitro and in animal models.
- SHEDs (Stem cells from Human Exfoliated Deciduous teeth): Higher proliferative capacity than DPSCs in culture.
- PDLSCs (Periodontal Ligament Stem Cells): Can regenerate cementum and periodontal ligament in animal xenograft models.
- SCAPs (Stem Cells from Apical Papilla): Located at developing root apices; important for root maturation.

These stem cells are governed by the same signaling pathways that are active during embryonic development. Wnt, BMP, and FGF manipulation, along with epigenetic modulation, can direct their differentiation in in vitro systems [11,12].

Although dental stem cells have shown promise in preclinical studies, few controlled clinical trials have been conducted. Shah et al. (2024) provided a comprehensive overview of the current understanding and future directions of dental stem cells in regenerative dentistry, highlighting that pulp regeneration trials using DPSCs have shown preliminary efficacy in limited patient series, but large-scale randomized controlled trials are lacking [13]. The transition from the bench to bedside remains a significant challenge.

### 7.3. Emerging and Experimental Approaches in Tissue Engineering

Emerging regenerative strategies, including whole-tooth bioengineering, dental organoid systems, and iPSC-based tooth regeneration, remain experimental and have not yet reached clinical applications. DPSCs seeded on hydrogels with growth factors have regenerated vascularized pulp-like tissue in animal models. Bioengineered tooth germs have successfully generated teeth in mice and larger animal models [63]. Quigley et al. (2024) recently reviewed tissue engineering approaches for dental pulp regeneration, highlighting the development of novel bioactive materials using pharmacological epigenetic inhibitors as a promising strategy to enhance regenerative outcomes [60].

Epigenetic modifications offer therapeutic potential because they are reversible. DNMT inhibitors, HDAC inhibitors, and miRNA mimics/inhibitors can modulate differentiation pathways in culture systems. However, significant challenges remain:

- Off-target effects of epigenetic modulators
- Delivery methods for clinical application
- Long-term safety profiles
- Scalability and cost considerations

Prioritized research roadmap

1. Near-term (1–3 years): Single-cell transcriptomic mapping of human dental development to identify cell populations and regulatory networks.
2. Medium-term (3–5 years): Development and validation of human iPSC-derived dental cell models and organoid systems for mechanistic studies and drug screening.
3. Long-term (5–10 years): Controlled clinical trials of stem cell-based therapies for pulp regeneration, periodontal regeneration, and potentially, whole tooth bioengineering. Recent breakthroughs in iPSC-derived dental organoids, such as the generation of human enamel-secreting ameloblast-like cells from iPSCs guided by single-cell transcriptomic atlases [64], underscore the feasibility of this approach.

## 8. Limitations of Current Evidence

The following are several important limitations to consider when interpreting the evidence reviewed herein:

Most mechanistic insights derive from mouse genetic models. Although mice share fundamental tooth developmental mechanisms with humans, significant differences exist in tooth number, morphology, and developmental timing. Additionally, mice have continuously growing incisors with persistent stem cell niches that do not exist in human teeth. Epigenetic regulation studies have largely relied on in vitro dental stem cell culture systems. The relevance of these findings to in vivo tooth development and regeneration requires further validation. While genetic associations between developmental genes and human dental anomalies have been well-established, direct evidence for signaling pathway functions during human odontogenesis is limited. Regenerative dentistry applications remain largely preclinical.

As with any narrative review, there is potential for selection bias toward positive results. Null and negative findings may be underrepresented in the published literature. Variations in cell isolation protocols, culture conditions, and differentiation assays across studies make direct comparisons challenging. Standardization of methodologies can strengthen the evidence base.

## 9. Conclusions

Tooth development exemplifies fundamental organogenesis principles, illustrating epithelial–mesenchymal interactions, morphogenetic signaling, and progressive cell fate specification. This review examined the integrated genetic and epigenetic regulatory mechanisms governing odontogenesis from initiation through eruption.

Wnt/β-catenin, BMP, FGF, and Shh pathways form an interconnected network that operates iteratively during development. These pathways converge on transcription factors, including PAX9, MSX1, PITX2, and LEF1, and execute stage-specific gene expression programs. Evidence for these mechanisms is derived primarily from mouse genetic models, with strong support from human genetic studies of dental anomalies.

Epigenetic mechanisms, such as DNA methylation, histone modification, and miRNA regulation, provide additional controls for precise spatiotemporal gene regulation. Specific miRNAs, including miR-21 (targeting STAT3), miR-140-5p (targeting Wnt1/β-catenin), and miR-218 (targeting DMP1), regulate dental stem cell differentiation in in vitro systems. However, the clinical translation of these findings requires further validation.

The clinical implications of this knowledge are still evolving. Genetic diagnosis for dental anomalies has now been clinically established. Dental stem cells offer cellular sources for potential regeneration; however, most applications remain in preclinical stages. Advances in tissue engineering combined with epigenetic modulation show promise for future tooth regeneration; however, significant translational gaps remain.

A key insight emerging from this review is that tooth development is governed not by individual pathways acting independently, but by a deeply integrated network in which genetic signaling, transcription factor activity, and epigenetic modifications operate as interconnected regulatory layers. Future studies should prioritize systems-level approaches, including single-cell multi-omics and spatial transcriptomics, to capture this complexity in its entirety and directly benchmark murine-derived mechanisms against human developmental programs [64,65]. In parallel, incorporating additional modulatory modules (e.g., EDA/EDAR/NF-κB, Notch, Hippo–YAP/TAZ, and RA signaling) will likely refine stage- and tissue-specific models and improve the design of regenerative perturbations [35,36,37,38,39,40,41,42,43].

Future research should prioritize the following: (1) single-cell and spatial approaches to comprehensively map the molecular landscape of human tooth development and cell–cell signaling niches [64,65]; (2) human iPSC-based models and organoid systems for mechanistic studies; and (3) controlled clinical trials to validate regenerative approaches. Only through rigorous clinical translation can the remarkable progress in understanding tooth development be realized as therapeutic benefits for patients.

## Figures and Tables

**Table 1:** Key transcription factors in tooth development and associated dental anomalies. Evidence levels: mouse = knockout/transgenic mouse models; human = clinical genetic studies with confirmed mutations. (Table reformatted for improved readability).

Transcription Factor	Primary Expression (Tissue/Stage)	Key Regulatory Role	Mouse Genetic Phenotype	Human Genetic/ Clinical Association	Key Refs.	Evidence Strength
PAX9	Dental mesenchyme; initiation–bud	Establishes mesenchymal odontogenic competence; supports progression beyond bud stage	Pax9−/−: tooth development arrested at bud stage; tooth agenesis	PAX9 variants: common cause of nonsyndromic tooth agenesis (often molars); genotype– phenotype correlations reported	[45]	Mouse KO + human LOF variants
MSX1	Dental mesenchyme; bud–cap	Cooperates with PAX9; acts downstream of BMP signaling to maintain odontogenic potential and patterning	Msx1−/−: arrested odontogenesis and cleft palate	MSX1 variants associated with tooth agenesis (sometimes with clefting)	[23,46]	Mouse KO + human LOF variants
PITX2	Dental epithelium; placode–bud	Earliest marker of odontogenic epithelium; regulates epithelial proliferation and epithelial–mesenchymal signaling	Pitx2 loss disrupts tooth morphogenesis (and other developmental processes)	PITX2 mutations: Axenfeld–Rieger syndrome with dental anomalies (agenesis, microdontia)	[21]	Mouse models + syndromic human mutations
LEF1	Dental epithelium/ enamel knot; bud–cap	Canonical Wnt effector; activates enamel-knot programs (e.g., FGF4) and links Wnt output to Shh regulation	Lef1−/−: bud-stage arrest	Direct LEF1 dental genotype–phenotype evidence remains limited; pathway-level human genetics supports canonical Wnt requirement	[28]	Mouse KO; limited direct human genetics

**Table 2:** Key microRNAs involved in odontogenic differentiation of dental stem cells. This table summarizes the principal miRNAs, their target genes, functional effects, and evidence levels as identified in recent systematic reviews.

miRNA	Reported Target(s)/ Pathway	Model System	Effect on Odontogenic Differentiation	Evidence Type (Representative)	Key Refs.	Evidence Strength
miR-21	STAT3 signaling axis (net pro- differentiation)	Human DPSCs	Promotes odontoblast differentiation and mineralization markers	miR-21/STAT3 axis interrogation (gain/loss-of-function)	[51,52,53]	Primarily in vitro; mechanistic axis defined
miR-27a	DKK3 and SOSTDC1 (Wnt/BMP)	Human DPSCs	Enhances odonto/ osteoblastic differentiation and mineralization	Mimic/inhibitor + luciferase validation; in vivo bone formation	[51,52,54]	In vitro + in vivo; translational relevance
miR-34a	NOTCH2 and HES1 (Notch signaling)	Human SCAPs	Promotes odontogenic/ osteogenic differentiation	Direct 3′UTR targeting + marker upregulation; Notch–miR feedback	[51,52,55]	Human stem cells in vitro; mechanistic target validation
miR-140-5p	Wnt1/β-catenin (net anti- differentiation)	Human DPSCs	Suppresses odontoblastic differentiation by reducing DSPP/DMP-1	Luciferase-validated targeting of Wnt1; mineralization assays	[51,52,56]	In vitro + target validation
miR-218	RUNX2 (net anti-osteogenic)	Human dental stem cells	Downregulated during mineralized differentiation; targets RUNX2	miRNA profiling during induction; functional targeting	[51,52,57]	Human cells; profiling + functional targeting
miR-143-3p	RANK (OPG–RANKL axis)	Human DPSCs	Inhibition enhances mineralization and upregulates DSPP/BSP/ ALP/OCN/OPN	Dual luciferase validation; differentiation markers + mineralization assays	[51,52,58]	In vitro + mechanistic pathway evidence

## Data Availability

No new data were created or analyzed in this study.
